# Pharmaceutical Board in Georgia

**Published:** 1881-12-20

**Authors:** 


### *PHARMACEUTICAL BOARD IN GEORGIA.*

The following are the gentlemen constituting the Board under the new law, to-wit:

Edward Barry, Augusta; John Ingalls, Macon; J. S. Pemberton, Atlanta; J. Zacharia, Columbus.

Physicians holding diplomas and have registered, are not required to undergo examination before the Board. It is sufficient to send their diplomas to any member of the Board, or to a friend who will attend to the matter for them.

